# Neuropsychiatric disorders following SARS-CoV-2 infection

**DOI:** 10.1093/brain/awad008

**Published:** 2023-02-02

**Authors:** Paul J Harrison, Maxime Taquet

**Affiliations:** Department of Psychiatry, University of Oxford, Warneford Hospital, Oxford OX3 7JX, UK; Oxford Health NHS Foundation Trust, Warneford Hospital, Oxford OX3 7JX, UK; Department of Psychiatry, University of Oxford, Warneford Hospital, Oxford OX3 7JX, UK; Oxford Health NHS Foundation Trust, Warneford Hospital, Oxford OX3 7JX, UK

**Keywords:** coronavirus, epidemiology, health records, memory, mental health

## Abstract

Several large-scale electronic health records studies have reported increased diagnostic rates for neuropsychiatric disorders following Coronavirus disease 2019 [COVID-19 or severe acute respiratory syndrome coronavirus 2 (SARS-CoV-2 infection)], but many questions remain. To highlight the issues, we selectively review this literature, focusing on mood disorder, anxiety disorder, psychotic disorder, and cognitive impairment (‘brain fog’). Eight key questions are addressed, comprising: (i) the nature and magnitude of the risks; (ii) their association with severity of infection; (iii) their duration; (iv) whether the risks differ between adults and children, or between men and women; (v) whether prior vaccination protects against them; (vi) the risk profile associated with different SARS-CoV-2 strains; (vii) what the underlying mechanisms might be; and (viii) whether the sequelae can be predicted. We consider the major unknowns, the limitations of electronic health records for research in this area, and the use of additional approaches to help characterize and understand the neuropsychiatric burden of COVID-19.

## Teaser

Harrison and Taquet review the literature on neuropsychiatric diagnoses following COVID-19, focusing on depression, anxiety, psychosis and cognitive impairment (brain fog). They address key issues including the magnitude of any increase in risk, and whether the risk profile differs between men and women or in association with different SARS-CoV-2 strains.

## Introduction

Early concerns that Coronavirus disease 2019 [COVID-19 or severe acute respiratory syndrome coronavirus 2 (SARS-CoV-2 infection)] might confer an increased risk of psychiatric and neurological disorders^1^ have largely been borne out.^2,3^ The findings have emerged from electronic health records (EHRs),^4–6^ claims databases,^7^ and self-report surveys,^8^ and are broadly consistent in reporting increased risks of many psychiatric and neurological symptoms and syndromes in the weeks and months after infection, as part of a broader post-acute COVID-19 syndrome.^9^ These risks are in addition to effects of the pandemic itself on mental health.^10–12^ However, many key questions about the links between COVID-19 infection and subsequent mental and brain health remain unanswered.

Here we address several of these questions, focusing on a limited number of disorders that highlight the issues: mood, anxiety and psychotic disorders, as well as cognitive impairment (‘brain fog’). Other neuropsychiatric sequelae of COVID-19 are covered elsewhere.^13,14^ We consider primarily the findings from our studies that have used the USA-based TriNetX Analytics EHR network (www.trinetx.com) to investigate risks for a broad range of psychiatric, neurological and cerebrovascular disorders. The value of EHRs in the context of the COVID-19 pandemic is that they provide large and up-to-date datasets with detailed demographic and biomedical data and standardized measures (e.g. ICD-10 diagnoses). EHR networks lend themselves to analyses whereby diagnostic rates after COVID-19 can be compared to matched cohorts experiencing other health events to generate relative risks as well as incidence figures—and with enough statistical power to look at subgroups, rare diagnoses, and time trends.

## What are the neuropsychiatric consequences of COVID-19 infection and how do the risks compare to other health events?

Several EHR studies have reported that, in the months after COVID-19, diagnoses of neuropsychiatric disorders as a whole, as well as for the major categories thereof, are commoner than is observed following other respiratory infections or health events. Taken from the largest study to date,^15^Table 1 shows the 6-month incidence of the diagnoses of interest after COVID-19 together with the hazard ratios (HRs) and absolute risk increases compared to a propensity score-matched contemporaneous cohort who had experienced another respiratory infection. The results show increased risks following COVID-19 infection for all the disorders, but with greater risks for psychotic disorder and brain fog (for definition, see Table 1 legend) than for mood and anxiety disorders. Increased risks for several other diagnoses were also seen after COVID-19,^15^ but not for eating disorders.^16^ Note that the findings in Table 1 (and all others we discuss here) are ‘first’ diagnoses, i.e. people who had no such diagnosis recorded before their infection. Incidences are of course higher, but HRs broadly similar, if recurrent diagnoses are included.^4,13^

**Table 1:** Selected neuropsychiatric disorders following SARS-CoV-2 infection

	Mood disorder	Anxiety disorder	Psychotic disorder	Brain fog
HR at 6 months versus other RTI (95% CI)	1.08 (1.06–1.11)	1.13 (1.11–1.15)	1.27 (1.18–1.37)	1.36 (1.33–1.39)
Incidence at 6 months (95% CI), %	2.95 (2.91–3.00)	4.98 (4.92–5.04)	0.20 (0.19–0.21)	2.65 (2.61–2.69)
Absolute risk increase at 6 months, %	0.15	0.37	0.036	0.64
Risk horizon, days	43	58	>730	>730
Time to equal incidence, days	457	417	>730	>730

Figures refer to first recorded diagnosis (i.e. people who had no diagnosis of that condition prior to infection). Incidence, hazard ratio (HR) and absolute risk increase are shown at 6 months compared to other respiratory tract infections (RTI). Risk horizon = the time at which the HR returns to 1. Time to equal incidence = the time when the cumulative number of diagnoses in the COVID-19 cohort is the same as the cumulative number of diagnoses in the comparator cohort. Diagnoses are ICD-10 codes: psychotic disorder (F20-F29), mood disorder (F30-F39), anxiety disorder (F40-F48). ‘Brain fog’ is a composite category^20^ of diagnostic codes for dementia (F01-F03, G30, G31.0, G31.83), mild cognitive impairment (G31.84), delirium (F05), encephalopathy (G93.40), and R codes for symptoms and signs of attentional or cognitive dysfunction (R40, R41, R48); the majority of people in the brain fog category had the R41 code, ‘other cognitive symptoms’.^15^ The 95% CIs are shown in parentheses. Data are from Taquet *et al*.^15,20^

The choice of comparator cohort (its inclusion criteria, and whether it is contemporaneous or historical) can affect HRs. However, elevated HRs, of comparable magnitude, are also seen when COVID-19 is compared to influenza and other health events,^4,6,13^ to uninfected contemporary controls,^5,7^ and to historical controls.^5,7,13^

Although statistically robust in large samples, the magnitude of increase of neuropsychiatric disorders after COVID-19 is limited, and the scale of the risks should not be exaggerated. For example, even for a common diagnosis like anxiety disorder, the absolute risk increase at 6 months after COVID-19 is only 0.37%, and psychotic disorder is only a 10th of that (Table 1). Similarly, Coleman *et al.*^6^ found a 0.8% absolute risk increase of any mental illness in the 12 months after COVID-19. Equally, these increases are not trivial in a public health sense: with over 650 million cases of COVID-19 to date, even an absolute risk increase of 0.1% translates to an additional 650 000 cases.

Three other studies have found less conclusive association between COVID-19 and subsequent neuropsychiatric disorder. A study using UK primary care records from 32 525 COVID-19 cases^17^ found no increased incidence of new-onset anxiety or depression after COVID-19 compared to other respiratory infections, though both were increased compared to the remaining population. However, strikingly low diagnostic rates were reported; for example, 0.05% of people were diagnosed with incident depression in the year after COVID-19 infection. Abel *et al*.^18^ also used UK primary care records and found no clear evidence that a positive SARS-CoV-2 test (*n* = 86 922) was followed by more incident depression and anxiety than with influenza or in those with a negative SARS-CoV-2 test. The study had a median follow-up of 6.3 weeks, and a composite of diagnoses and symptoms was measured. In the Danish population, Lund and colleagues^19^ reported that non-hospitalized SARS-CoV-2 positive people (*n* = 10 498) were not at increased risk of anxiety or depression diagnoses in the following 6 months compared to test-negative people. However, only hospital-based diagnoses were recorded, and some unexpected results found (e.g. pulmonary disease and cough being markedly less likely in the SARS-CoV-2-positive cohort).

Beyond the individual features of these three studies, there are other potential explanations to account for their differing conclusions compared to the ‘positive’ EHR studies. Firstly, the latter are larger, ranging from 153 000 cases^5^ to 1.28 million cases,^15^ thus increasing statistical power. Indeed, the smaller negative studies report confidence intervals (CIs) that often include the effect sizes observed in the larger positive ones. Secondly, the positive studies are US-based whilst the negative ones are from the UK and Denmark, and health care provision and the organization of health records differ between these countries. For example, the European studies are weighted strongly towards primary care data and are therefore more likely to capture milder or asymptomatic COVID-19. This approach provides more accurate estimates of incidences in the general population of patients infected with COVID-19, but possibly biases relative risks, depending on how the comparator cohorts are defined and identified.

Overall, differing results between studies according to methodology and denominator populations are therefore not unexpected, and should be viewed accordingly. Equally, the largest EHR studies do paint a clear picture of a modestly but significantly increased risk of neuropsychiatric disorders in the months after COVID-19 infection. The following questions are addressed primarily with regard to the US EHR literature, and we acknowledge that some conclusions—and questions—might be different if the focus were on other studies and study designs.

## Are neuropsychiatric risks related to the severity of the infection?

COVID-19 can range from an asymptomatic infection to a fatal condition, and an important question is whether the severity of the infection is related to the risk of various sequelae. To answer this question, and to avoid the various sources of confounding noted in the previous section, we limit our considerations here to studies which make direct comparisons between severity-based subgroups. Hospital admission is the commonest proxy marker of severity and has been examined in three EHR studies.^5,7,13^ These are consistent in finding that hospitalization is associated with greater risk, but that non-hospitalized patients are also at elevated risks of subsequent neuropsychiatric disorders. Both these findings are important. First, the latter shows that risks are not limited to the subgroup who experienced an infection of sufficient severity to require admission, but also apply to the larger number of people who did not. Second, the finding that disorders are more likely in patients who were hospitalized implies a relationship between severity of infection and degree of risk.

The impact of COVID-19 on neuropsychiatric outcomes is less clear for infections severe enough to have required intensive care unit (ICU) admission. COVID-19 patients admitted to ICU have a higher incidence and greater risk of these disorders compared to matched COVID-19 patients not admitted to ICU (Table 2).^13,20^ However, neuropsychiatric disorders are common after ICU admission for any cause^21,22^ and it is not known whether the risks differ in post-ICU COVID-19 patients compared to other post-ICU patients.^23^ Similarly, although delirium is associated with a markedly higher risk of neuropsychiatric diagnoses after COVID-19 than in non-delirious COVID-19 patients,^13^ it is not clear if these risks are greater than those for delirium in other acute contexts. Answering these questions is important not only to prognosticate risks but also to delineate better the specificity of post-COVID-19 sequelae.

**Table 2:** Relationship of post-COVID-19 neuropsychiatric disorders with hospitalization and ICU admission

	Mood disorder	Anxiety disorder	Psychotic disorder	Brain fog
**Hospitalized versus non-hospitalized COVID-19**				
ȃHR at 6 months (95% CI)	1.53 (1.33–1.75)	1.49 (1.34–1.65)	2.77 (1.99–3.85)	2.24 (2.12–2.35)
ȃIncidence in hospitalized cases at 6 months, %	4.49 (4.05–4.99)	6.91 (6.38–7.47)	0.89 (0.72–1.09)	16.66 (16.14–17.17)
ȃIncidence in non-hospitalized cases at 6 months, %	3.86 (3.60–4.14)	6.81 (6.47–7.16)	0.25 (0.19–0.33)	4.91 (4.71–5.11)
**ICU versus non-ICU COVID-19**				
ȃHR at 6 months (95% CI)	2.06 (1.57–2.71)	2.22 (1.82–2.71)	1.77 (0.98–3.20)	2.54 (2.32–2.74)
ȃIncidence in ICU cases at 6 months, %	5.82 (4.86–6.97)	9.79 (8.65–11.06)	0.70 (0.46–1.06)	27.75 (26.58–28.91)
**Non-hospitalized COVID-19 versus non-hospitalized other RTIs**				
ȃHR at 6 months (95% CI)	1.37 (1.27–1.47)	1.37 (1.30–1.45)	1.49 (1.15–1.93)	N/A

Incidence and HR in hospitalized versus non-hospitalized COVID-19 (*n* = 45 167 in each cohort, except *n* = 52 597 for brain fog); in cohorts of COVID-19 admitted or not admitted to ICU (*n* = 8942 in each cohort), and in non-hospitalized COVID-19 versus non-hospitalized other respiratory infections (RTIs; *n* = 183, 731 in each cohort). Matched cohorts used for HRs; complete cohort used for incidences. See Table 1 legend for diagnostic categories. Data from Taquet *et al*.,^13^ except for brain fog.^20^ These studies excluded children younger than 10 years old.

## How long do the risks last?

If the risk of neuropsychiatric disorders is increased after COVID-19 infection, a key question—for patients and for health services—is the trajectory and duration of these risks. With the increasing size and duration of the pandemic, this is now becoming a tractable question and has been addressed using time-varying HRs in people up to 2-years after infection.^15^ This study reported the ‘risk horizon’, i.e. the time after which there is no greater risk of that diagnosis being made in the COVID-19 cohort than in the comparator cohort. It also reported the ‘time to equal incidence’, which is the time when the cumulative incidence is the same in the two cohorts and at which there is therefore no overall greater burden from COVID-19. Note that a time to equal incidence can only be reached if, after the risk horizon, the risk becomes higher in the comparator cohort than in the COVID-19 cohort (i.e. the HR falls below 1).

Table 1 shows that these parameters differ markedly between outcomes. Mood and anxiety disorders show a transient trajectory, with a risk horizon of less than 2 months and a time to equal incidence of about 15 months. In contrast, psychotic disorder and brain fog had not reached a risk horizon, nor therefore a time to equal incidence, within 2 years, indicating an ongoing greater risk after COVID-19. Note that the timings refer to the date of diagnosis made in the EHR, not the onset of symptoms, which may have predated this by weeks or months. Thus, the trajectory of risk for onset of symptoms after COVID-19 infection may be less prolonged than implied by the risk horizon; this is currently unknown.

## Do age or gender influence the neuropsychiatric risks?

The results in Table 1 are pooled estimates across the lifespan, and most other large-scale studies have either been restricted to adults or a specific age group (e.g. the over-65s).^24^ In a recent study,^15^ analyses were conducted in children, adults and older adults, revealing age-related differences as well as similarities in the relationship between COVID-19 and psychiatric disorders (Table 3). In contrast to adults, children were not found to be at greater risk of mood and anxiety disorders after COVID-19, and though children are at greater risk of psychotic disorders and brain fog, the risk horizons are shorter than for adults and older adults. For brain fog, a time to equal incidence is reached (after about 16 months), indicating that the total number of cases is ultimately equal between children diagnosed with COVID-19 and those with another respiratory infection. These findings in children are largely reassuring, especially since the incidence of each disorder is much lower than in adults, but equally, they show that children are not immune to neuropsychiatric consequences of COVID-19. Moreover, children are at greater risk than adults of seizures and epilepsy following COVID-19.^25^ Adults and older adults were similar in most respects, although the latter group tended to have higher incidences and HRs, particularly for cognitive impairment.^15,20^

**Table 3:** Risks of neuropsychiatric disorders after COVID-19 across the lifespan

	Mood disorder	Anxiety disorder	Psychotic disorder	Brain fog
**HR at 6 months (95% CI)**				
ȃChildren	1.02 (0.94–1.10)	1.00 (0.94–1.06)	2.00 (1.26–3.19)	1.20 (1.09–1.33)
ȃAdults	1.06 (1.04–1.09)	1.13 (1.11–1.15)	1.18 (1.08–1.29)	1.35 (1.31–1.40)
ȃOlder adults	1.17 (1.11–1.22)	1.16 (1.11–1.20)	1.39 (1.21–1.59)	1.41 (1.36–1.46)
**Incidence at 6 months, % (95% CI)**				
ȃChildren	1.50 (1.41–1.59)	2.63 (2.51–2.75)	0.04 (0.029–0.052)	1.02 (0.95–1.09)
ȃAdults	3.20 (3.14–3.26)	5.62 (5.54–5.71)	0.20 (0.18–0.21)	2.09 (2.05–2.13)
ȃOlder adults	3.20 (3.09–3.30)	4.68 (4.55–4.81)	0.31 (0.28–0.34)	5.98 (5.84–6.11)
**Absolute risk increase at 6 months, %**				
ȃChildren	−0.054	−0.13	0.018	0.11
ȃAdults	0.11	0.44	0.022	0.52
ȃOlder adults	0.38	0.43	0.088	1.56
**Risk horizon, days**				
ȃChildren	–	–	75	75
ȃAdults	44	65	86	>730
ȃOlder adults	36	44	>730	>730
**Time to equal incidence, days**				
ȃChildren	–	–	>730	491
ȃAdults	330	466	657	>730
ȃOlder adults	>730	463	>730	>730

Table shows incidence, HR, absolute risk increase, risk horizon and time to equal incidence for selected psychiatric disorders in children (<18 years; *n* = 185 748), adults (18–64 years; *n* = 856 588) and older adults (≥65 years; *n* = 242 101). See Table 1 legend for diagnostic categories. Data from Taquet *et al*.^15^

Only a few studies have investigated whether men and women differ in their risks of neuropsychiatric disorders after COVID-19, and results are unclear. In a predominantly male Veterans Administration dataset, the HR for mental health disorders between patients with COVID-19 and the rest of the population was greater in males than females.^14^ Another study directly comparing matched females and males who had had COVID-19 found that women were at a higher relative risk of anxiety/depression than men, but at a lower risk of brain fog.^20^ In contrast, another study of COVID-19 patients reported no gender difference in risk for mental health disorders or memory difficulties.^7^ Gender, like race and ethnicity, deserves greater attention in future studies of neuropsychiatric disorders after COVID-19.

## Does vaccination affect neuropsychiatric risks following a breakthrough infection?

The benefits of vaccination against severe or fatal COVID-19 infection are unequivocal, and hence the best way to prevent all sequelae of COVID-19 is vaccination. However, it is less clear whether vaccination reduces the incidence of neuropsychiatric disorders in people who experience a breakthrough infection (i.e. a COVID-19 infection after being vaccinated). This becomes an increasingly important question as this population now represent a large (and increasing) fraction of all COVID-19 infections. Al-Aly *et al*.^26^ found a reduced risk of a composite mental health outcome in vaccinated cases, based on 33 940 breakthrough infections [HR = 0.85 (0.79–0.92)]. Another study, of 10 024 breakthrough infections, found a lower incidence of psychotic disorder [HR = 0.65 (0.52–0.79)], brain fog [HR = 0.87 (0.76–0.99)], and many other adverse outcomes, in the post-vaccination cases, but no difference in incidence of mood disorder [HR = 1.03 (0.92–1.14)] or anxiety disorder [HR = 1.00 (0.90–1.10)], compared to matched unvaccinated cases.^27^

These studies indicate that post-vaccination breakthrough COVID-19 infections are associated with a reduction in risk of neuropsychiatric diagnoses, but the magnitude and profile of protection remains unclear.^28^ It is not known whether different vaccines or vaccine schedules influence these outcomes, although greater protection was seen for people who had received two doses rather than one.^27^ It should also be noted that many vaccinations may be unrecorded in EHRs; thus, some people assigned to a non-vaccinated COVID-19 group may in fact be vaccinated, tending to bias HRs to the null. As with SARS-CoV-2 infection itself, the extent of this under-recording may vary between populations and across time.

## Do SARS-CoV-2 strains lead to similar neuropsychiatric risks?

Isolating effects of SARS-CoV-2 strain on risks of neuropsychiatric outcomes is difficult, since it is conflated by temporal trends in vaccination rates, vaccine types and other time-varying effects of the pandemic. It is also complicated by differential mortality rates during the acute infection. Large scale studies also lack individual serotyping. Taquet and colleagues^15^ attempted to minimize these limitations by comparing matched individuals who survived a COVID-19 infection in time windows just before compared to just after emergence of a dominant strain—Alpha, Delta and Omega. Alpha had similar neuropsychiatric outcomes as the original strain. Delta (compared to Alpha) was associated with an increased risk of brain fog and anxiety disorder, and equivocally psychotic disorder, but with a similar risk of mood disorder. While it led to a much less severe acute illness, Omicron had comparable risks to Delta for all these disorders. The findings suggest that the risk of post-COVID-19 neuropsychiatric disorders may continue with future strains that may also, like Omicron, be milder in other respects—although the partial protection provided by vaccination noted earlier will likely reduce this burden to some extent.

## What mechanisms explain the association between COVID-19 and subsequent neuropsychiatric disorder?

Several mechanisms have been proposed to explain the neuropsychiatric sequelae of SARS-CoV-2 infection.^11,29,30^ These include direct viral invasion, autoimmune processes, peripheral or central inflammation, and cerebrovascular impairment related to microthrombi and endotheliopathy. And, for mental health outcomes, psychological (e.g. the stress of being infected with a new virus and its unknown consequences) and psychosocial (e.g. the disrupted support network or the loss of income caused by the need to self-isolate) explanations must also be considered. The occurrence and relative contributions of these mechanisms remains unclear, but some initial clues are beginning to emerge.

SARS-CoV-2 enters endothelial cells lining small cerebral vessels, and its main protease (M^pro^) cleaves nuclear factor-κB essential modulator and induces cell death.^31^ A damaged endothelium is likely to be followed by microthrombus formation and other adverse events including microglial activation.^32^ Wenzel and colleagues^31^ showed that inhibition of the receptor-interacting protein kinase (RIPK) signalling pathway prevented the M^pro^-induced effect. Notably, phenytoin is known to have RIPK-inhibiting properties, and pharmacoepidemiological evidence indicates that people taking phenytoin at the time of their COVID-19 infection are at lower risk of cognitive impairment, supporting the hypothesis that the latter is due at least in part to an endotheliopathy-related mechanism.^33^ This finding needs to be complemented by empirical data. For example, showing elevation of biomarkers predicted to be raised in this scenario, or by neuroimaging and neuropathological correlates of microvascular pathology.^34–36^ A neuroinflammatory mechanism, marked by high C-C motif chemokine 11 (CCL11) levels in CSF, may also contribute to post-COVID cognitive deficits via CCL11-mediated microglial activation.^37^ Regardless of the mechanism(s), it remains to be explained why the increased risk of a new diagnosis of brain fog (Table 1) and dementia^15^ persists for up to 2 years after COVID-19 infection. It is possible that a neuroinflammatory or microvascular process becomes chronically activated,^35,38^ or the ongoing risk may require additional downstream events, or may simply reflect delayed diagnosis.

The common psychiatric disorders—depression and anxiety—show transient risk, and with no excess risk within 2 years of COVID infection (Table 1). This short-lived risk profile suggests that COVID-19 might have precipitated, or brought forward, disorders that otherwise would have occurred later, and might be interpreted within the psychological or psychosocial framework mentioned earlier. The finding that SARS-CoV-2-positive people who were unaware of having had the infection do not show an association with subsequent mental health sequelae is consistent with this possibility.^39^ Clearly, explanations of this kind are simplistic, and encourage a false dichotomy between ‘organic’ and ‘functional’. However, they draw attention to the need for a broad range of explanatory models, and highlight that no one mechanism is likely to be relevant to the range of neuropsychiatric disorders associated with COVID-19 infection.

## Can we predict who is at risk of post-COVID-19 neuropsychiatric disorders?

It is worth emphasizing that most people do not develop a post-COVID-19 neuropsychiatric disorder, including those who had severe infection (Table 2). Even the disorders with high incidences have low absolute risk increases, indicating that a large proportion of cases cannot be attributed to COVID-19 (Table 1). At present little is known about predicting those who will be affected, beyond the demographic factors discussed here such as age, hospitalization and prior vaccination. Various biomarkers have been reported to associate with persistent symptoms or impairments after COVID-19,^3,40,41^ but other than the CCL11 findings mentioned^37^ we are not aware of robust predictors of neuropsychiatric disorders. This is a critical research question, and is being addressed in longitudinal cohort studies such as PHOSP-COVID^42^ and RECOVER.^43^

## What are the limitations and gaps in the data?

We have emphasized findings arising from our own EHR studies, in part because they are amongst the largest and with the longest duration of follow up, allowing more traction on the key issues discussed above. However, by the same token, many of the results await replication and thus should be viewed with caution. Moreover, all EHR studies have limitations.^13,44,45^ Most of the limitations are generic, of which the most important is arguably the fact that EHRs, like surveys, provide observational data, and therefore cannot prove or disprove causality nor readily identify mechanisms explaining associations (such as those between COVID-19 and health outcomes). Some limitations are particularly pertinent to the use of EHRs to study effects of COVID-19.^46^ These are summarized in Table 4, with reference to their potential impact on estimates of relative risk and incidence of post-COVID-19 neuropsychiatric disorders. Although the actual effect of these factors remains unclear, and may well be limited, the various limitations incentivize other investigational designs, such as the longitudinal cohort studies mentioned above.

**Table 4:** Some limitations of electronic health records for COVID-19 research, and how they may impact on the relative risk and incidence of neuropsychiatric disorders

Limitation	Effect on relative risk of NPD	Effect on incidence of NPD after COVID-19	Comments and implications
Undocumented COVID-19 infection (in comparator cohort)	Decreased	Unaffected	Some subjects in comparator cohorts will have had COVID-19
People with asymptomatic COVID-19 under-represented	Unaltered if comparator cohort has a similar limitation (e.g. other respiratory infection). Increased if comparator cohort does not have a similar limitation (e.g. general population or all people testing negative for COVID-19) **and** if NPDs are less common after asymptomatic COVID-19	Increased, if NPDs are less common after asymptomatic COVID-19	Using multiple comparator cohorts can help better characterize relative risk. Prospective studies specifically targeting asymptomatic COVID-19 cases can help determine the incidence of NPDs in that subgroup.
NPD might have started before the index event but not diagnosed till afterwards	Unlikely	Increased	Could lead to misattribution of causality to COVID-19 or other index event
NPD present but not yet diagnosed (e.g. because of diagnostic delay or because affected individual does not seek medical attention)	Unlikely	Decreased	The effect on incidence will tend to counter the over-estimation arising from the limitation above. Active follow-up in prospective studies is required to detect these cases
Incomplete past health records	Unlikely	First-onset diagnostic rates increased if previous episodes had occurred but were not recorded	Less of a problem in countries where people have a single health record
Lack of information on viral strain	Could be increased, decreased or unchanged	Could be increased, decreased or unchanged	Effects depend on whether the strain affects risk of NPD and, if so, the timing and design of the study
SARS-CoV-2 could affect patient or physician behaviour and thence diagnostic behaviour	Could be increased, decreased or unchanged	Could be increased, decreased or unchanged	Direction of effect depends on what (if anything) COVID-19 does to health-seeking or physician behaviour. Difficult limitation to overcome.
People making no further contact with health services considered lost to follow-up	Could be increased, decreased, or unchanged	Decreased when using Kaplan–Meier estimator, unchanged when using total counts	Prospective studies with active follow-up and low attrition can address this limitation
Unknown NPD severity, course, and outcome	None	None	Difficult to assess using conventional EHR data. Although risk and incidence unaffected, these factors have implications for treatment and services

NPD = neuropsychiatric disorder.

## Conclusions

From large-scale EHR studies, associations between COVID-19 infection and an increased risk of neuropsychiatric disorders appear robust. However, as the incomplete answers to the questions posed above indicate, much remains to be determined. More studies, in different populations and health care settings, will help fill in the gaps and reveal the longer-term trajectory, the factors which mediate or modify the associations, and the ways in which neuropsychiatric outcomes after COVID-19 differ from those after other viral infections. They can also explore the risks associated with COVID-19 reinfection.^47^ However, increasingly the priority is to identify the mechanisms and predictors. It is only through their discovery that rational interventions to treat or prevent the neuropsychiatric burden of COVID-19 can be designed and tested.
